# Unlocking the Non-invasive Assessment of Conduit and Reservoir Function in the Aorta: The Obstructive Descending Aorta in HLHS

**DOI:** 10.1007/s12265-022-10221-4

**Published:** 2022-02-23

**Authors:** Adelaide de Vecchi, Alessandro Faraci, Joao Filipe Fernandes, David Marlevi, Hannah Bellsham-Revell, Tarique Hussain, Nidhin Laji, Bram Ruijsink, James Wong, Reza Razavi, David Anderson, Caner Salih, Kuberan Pushparajah, David Nordsletten, Pablo Lamata

**Affiliations:** 1 School of Biomedical Engineering and Imaging Sciences, King’s College London, 5th Floor Becket House, Lambeth Palace Road, London, SE1 7EU UK; 2 Massachusetts Institute of Technology, Cambridge, MA USA; 3 Department of Congenital Heart Disease, Evelina London Children’s Hospital, Guy’s & St Thomas’ Hospitals, London, SE1 7EH UK; 4 Pediatric Cardiology, UT Southwestern, Children’s Medical Center Dallas, 1935 Medical District Dr, Dallas, TX 75235 USA; 5 Department of Biomedical Engineering and Cardiac Surgery, University of Michigan, Ann Arbor, MI USA

**Keywords:** Aorta, Reservoir function, Conduit function, 4D flow, Hypoplastic left heart syndrome

## Abstract

Aortic surgeries in congenital conditions, such as hypoplastic left heart syndrome (HLHS), aim to restore and maintain the conduit and reservoir functions of the aorta. We proposed a method to assess these two functions based on 4D flow MRI, and we applied it to study the aorta in pre-Fontan HLHS. Ten pre-Fontan HLHS patients and six age-matched controls were studied to derive the advective pressure difference and viscous dissipation for conduit function, and pulse wave velocity and elastic modulus for reservoir function. The reconstructed neo-aorta in HLHS subjects achieved a good conduit function at a cost of an impaired reservoir function (69.7% increase of elastic modulus). The native descending HLHS aorta displayed enhanced reservoir (elastic modulus being 18.4% smaller) but impaired conduit function (three-fold increase in peak advection). A non-invasive and comprehensive assessment of aortic conduit and reservoir functions is feasible and has potentially clinical relevance in congenital vascular conditions.

**Graphical abstract:**

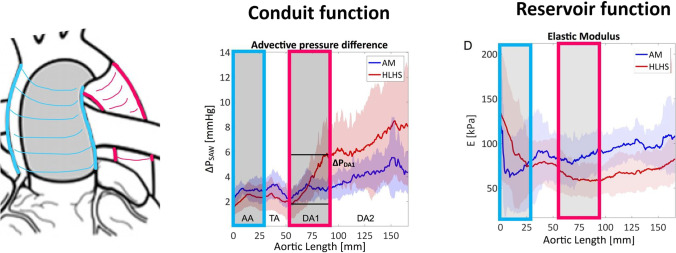

**Supplementary Information:**

The online version contains supplementary material available at 10.1007/s12265-022-10221-4.

## Introduction

The large arteries, and in particular the aorta, have two main functions: they serve as a conduit to transport the blood from the ventricles to the body and as a reservoir of blood that fills during systole and recoils during diastole (i.e. Windkessel function) to reduce pulsatility towards the capillaries. The conduit function depends on the vessel calibre, on its ability to accommodate the net blood flow and the lack of obstructions — its assessment is based on pressure differences across the vessel that are obtained either from invasive catheterised sensors or velocity derived surrogates, most conventionally from Doppler echocardiography. The reservoir function depends on the mechanical compliance of the vessel wall and its ability to respond to flow and pressure changes — its assessment is based on the pulse wave velocity (PWV) or on the distensibility of the artery (i.e. the change of radius over the cardiac cycle) that are obtained from skin tonometry sensors or imaging studies [1].

Hypoplastic left heart syndrome (HLHS) is a congenital condition that requires staged surgical palliation, with the first step performed immediately after birth. The aim at this first stage is to restore the conduit function, i.e. to correct any coarctation or hypoplasia and to create a new aortic conduit (neo-aorta) able to accommodate the entire ventricular output [2]. This requires the reconstruction of the ascending aorta and transverse arch using the pulmonary artery with anastomosis of the native aorta (Damus-Kaye-Stansel, or DKS, anastomosis). This surgical augmentation of the conduit uses homograft material that is known to reduce the reservoir function due to its increased stiffness [3, 4]. A study of the compromise between the two aortic functions, conduit and reservoir, is thus desirable to understand the impact of surgical interventions in HLHS and to optimise their approach. Such study is further motivated by the fact that, despite numerous HLHS patients now surviving staged palliation, the right ventricle (RV) and circulation are likely to fail over time, and unfavourable haemodynamic conditions due to abrupt changes in stiffness, curvature and diameter of the reconstructed vessel contribute to this risk by increasing ventricular afterload. There is also increasing evidence that significant neurodevelopmental issues associated with HLHS may be triggered by these conditions [5].

Recent advances in medical imaging and digital twin technology [6] now enable a detailed analysis of the aortic anatomy and function, including non-invasive estimation of blood pressure differences via 4D flow reconstruction from phase-contrast magnetic resonance imaging (PC-MRI) [7–10]. These techniques have been validated against catheter measurements both in vitro and in vivo in healthy volunteers and in patients with aortic stenosis and coarctation [11, 12]. Building on the opportunities offered by these technologies, this study proposes a non-invasive method to assess the conduit and reservoir function in infant arteries and presents its application to the reconstructed HLHS aorta, where understanding the relationship between anatomy and function is crucial to assess outcomes.

## Methods

The proposed method to assess conduit and reservoir function of an artery is based on an acquisition of a dense velocity field (e.g. from 4D flow MRI in our study). The conduit function is assessed by two metrics, the pressure difference caused by flow advection (i.e. by the spatial acceleration needed to accommodate the flow through a given vessel calibre) and the viscous energy dissipation. The reservoir function is assessed by the elastic modulus (E) derived from the PWV that is estimated from temporally sparse but spatially dense velocity vectors available from 4D flow MRI. The method is used to compare the surgically reconstructed aortas from HLHS subjects to the native aortas of age-matched controls.

### Patient Population and Surgical Procedure

Data from 10 pre-Fontan HLHS patients (median age: 2.70 years, *IQR*: 2.43 to 3.48 years, baseline characteristics in Table 1) and 6 age-matched (AM) patients (median age: 2.48 years, *IQR*: 2.1 to 2.80) with non-reconstructed aortic anatomy were retrospectively compared. The reconstruction was performed at our centre (Evelina London Children’s Hospital, London, UK). Patients were imaged to assess function and anatomy prior to the final stage of palliative surgery. The recruitment period spanned approximately 10 months. The HLHS cohort included 3 cases of hybrid procedure and 7 cases of primary classical Norwood procedure. The median sizes of the ascending aorta on initial echo ranged from 2.5 mm in patients with mitral and aortic atresia to 4.2 mm in cases of mitral and aortic stenosis (details at SM1, Supplementary Material). DKS anastomosis was performed with a side-to-side between the native aortic root and adjacent pulmonary artery. A homograft patch was then used to construct an augmented ascending aorta and transverse arch reaching a counter incision on the descending aorta. The AM control group consisted of other congenital conditions where the aorta did not require reconstruction (details at SM1, Supplementary Material).

**Table 1:** Baseline patient characteristics from MRI data

	HLHS (*n* = 10)
Sex, M/F	7/3
Age (years)	2.8 ± 0.7
Body surface area (m^2^)	0.57 ± 0.09
Cardiac output (l/min/m^2^)	4.75 ± 0.79
EFF (ml/beat/m^2^)	55.3 ± 8.4
EF (%)	63.6 ± 7.7
Neo AV area (mm^2^)	402.0 ± 103.4
AA area, RPA level (mm^2^)	411.9 ± 172.6
ESV (ml)	19.2 ± 7.1
EDV (ml)	76.4 ± 26.6
Heart rate (bpm)	86 ± 14

Values in *n* or *mean* ± *SD*. *EF*, ejection fraction; *EFF*, effective forward flow; *AV*, aortic valve; *AA*, ascending aorta; *RPA*, right pulmonary artery; *ESV*, end systolic volume; *EDV*, end diastolic volume

This study was approved by the local ethics committee (08/H0810/058) at Evelina Children’s Hospital, London (UK). All data were acquired after informed consent was received from all subjects involved, in accordance with the Declaration of Helsinki.

### Image Acquisition

MRI data were obtained using a SENSE acquisition on a Philips 1.5-Tesla Achieva scanner (Philips Healthcare, Best, Netherlands). Anatomical data included 3DSSFP sequences acquired using a respiratory navigator following intravenous injection of contrast agent. Patients were given 0.1 mmol/kg body weight of either gadopentetate dimeglumine (Magnevist, 41 Berlex Laboratories, Wayne, NJ, USA) or gadoterate meglumine (Dotarem, Guerbet, Villepinte, France). An acceleration factor of 2 was employed with a flip angle of 40° and a breath-hold time 20–30 s. Images had 1.2–1.7-mm isotropic voxel size.

Full-field aortic blood flow was acquired from a free-breathing, prospectively ECG-triggered PC-MRI sequence with velocity encoding of 120 cm/s. A spatial resolution of 2.0-mm isotropic voxels and a temporal resolution below 35 ms (corresponding to 24–30 phases) were employed (mean field of view 300 × 70 × 150 mm; *TR* = 3.8 ms; *TE* = 2.4 ms; flip angle 5°; acceleration kt + , 8; and bandwidth = 500 Hz). Respiratory gating for motion correction was applied and data were reconstructed using an in-house implemented kt-principal component analysis method [9, 13]. Automatic eddy current correction was applied to all data.

### Anatomical Analysis

Anatomical measurements were derived from 3DSSFP MRI data and a virtual angiography generated from the PC-MRI images at peak systole.

Wall thickness was computed from the 3DSSFP MRI data as the average difference between the systolic endo- and epi-vascular diameters in a cross section perpendicular to the aortic centreline, averaging the measurements by three independent observers (SM3, Supplementary Material).

**Fig. 1:**
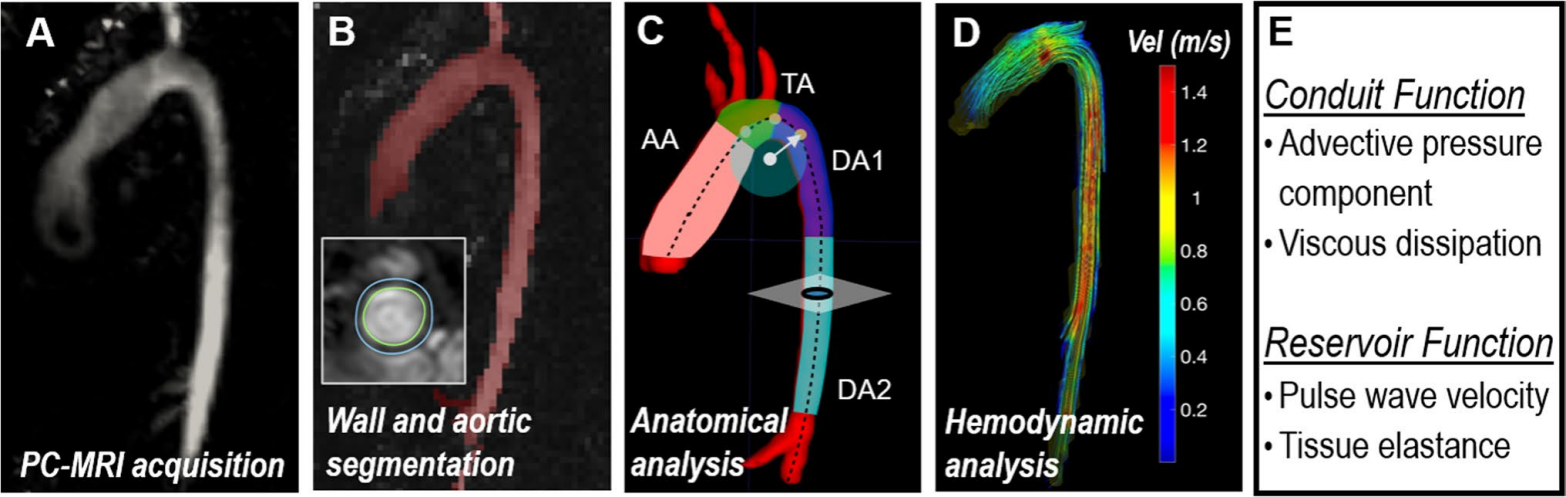
Methodological overview to extract the conduit and reservoir functions of a vascular segment. **A** Acquisition of 4D flow MRI data. **B** Anatomical segmentation from images of peak systolic velocity magnitude. **C** Subdivision of the aorta into 4 segments and extraction of shape metrics (centreline, curvature, diameter). **D** Flow velocity vectors and magnitude iso-contours reconstructed from PC-MRI data. **E** Extraction of descriptors of conduit and reservoir function

The rest of the analysis was based on the virtual angiography, and as such measurements correspond to peak systolic events and do not account for the changes throughout the cardiac cycle. Aortas were divided into four segments (Fig. 1): AA, TA and upper and lower thoracic descending aorta (DA1 and DA2, respectively). These segments were manually identified based on landmarks, i.e. aortic valve plane, brachiocephalic artery, left subclavian artery, point at which the descending aorta (DA) matches aortic valve level and diaphragm level. Aortic curvature was quantified by the inverse of the radius of the circumference defined by three consecutive points along the centreline (SM4, Supplementary Material). Aortic diameter was obtained from the cross-sectional area at a given perpendicular plane to the centreline. The diameter and curvature in each segment were obtained by averaging all measurements along the centreline at 1-mm intervals. All length measurements were indexed by body surface area for population uniformalisation.

### Underpinning Theory for Conduit Function: the Components of the Pressure Differences

The conduit function of the aorta is assessed by the pressure differences along the aorta, with larger differences indicating higher resistance to the flow and hence a worse conduit function. 4D flow data allows for a comprehensive and non-invasive estimation of pressure differences applying the 3D Navier–Stokes equations [7, 14–16]. In essence, the pressure changes driving the flow are the result of three types of energy contributions, i.e. the temporal variations in kinetic energy ($\frac{\partial {K}_{e}}{\partial t}$), the advective energy (*A*_e_) and the viscous energy (*V*_e_) [8], but only two of these contributions will be considered to evaluate the conduit function, as explained next.

In more detail, $\frac{\partial {K}_{e}}{\partial t}$ quantifies the temporal variation in the blood flow momentum, i.e. the pressure changes due to the blood acceleration and deceleration in time. This component causes the pressure in the DA to be larger than in the AA during the second half of systole, when blood is being decelerated. A larger or smaller $\frac{\partial {K}_{e}}{\partial t}$ will be mainly related to the inotropic status of the heart, its contractile ability. Besides, the net contribution of this component during a heart cycle is small since the acceleration and deceleration parts cancel each other, reason why this component is not accounted for when assessing the stenotic burden in clinical practice [14]. These characteristics of the temporal variations of blood momentum, which is an important part of the pressure differences along a vascular segment [8], justify its exclusion in the assessment of the conduit function of a vessel.

The *A*_e_ component is indeed relevant for the characterisation of the conduit function and obstructions since it captures the effect of spatial acceleration caused by a change in lumen width [14]. When a vessel narrows its calibre, a sudden variation in *A*_e_ causes a positive pressure difference that accelerates the blood through the narrower orifice — and vice versa in a widening vessel. *A*_e_ is thus the functional signature of changes in vessel calibre, such as tapering or coarctation, and will be the focus in this study.

Finally, *V*_e_ accounts for the energy dissipation due to friction, and therefore it is a metric for haemodynamic inefficiencies. Viscous dissipation can be laminar (i.e. friction between ordered layers of flow) or turbulent (i.e. friction between irregular fluctuations). While the assessment of the turbulent component requires specialised 4D flow sequences [15], the laminar effects, available from conventional 4D flow sequences, will be quantified in this study and reported as *V*_e_ rate [17].

These concepts are expressed mathematically by the work-energy relative pressure (WERP) formulation, which states that the total pressure difference along a vessel is based on the three energy contributions described above [7]:

1 $${\Delta p}_{\mathrm{tot}}= \frac{1}{Q}\left(\frac{\partial {K}_{\mathrm{e}}}{\partial t}+{A}_{\mathrm{e}}+{V}_{\mathrm{e}}\right)$$

To obtain the corresponding pressure variation, each of the energetic components is divided by the flow rate through the aorta, *Q*, which is the integral across the aortic cross section.

### Assessment of Conduit and Reservoir Function

Blood velocity vectors are reconstructed from 4D flow data (Fig. 1D) and used to characterise the conduit and reservoir functions.

The conduit function is assessed by the advective pressure component and by the laminar viscous energy rate across a vascular segment. Both physical magnitudes are studied at peak systole, when their impact is the greatest, and related to the total pressure differences during the heart cycle obtained with the WERP formulation [7]. The advective pressure component is computed using the simplified advective WERP formulation (SAW) [14] along the vascular segment — SAW is conceptually a correction of the commonly used simplified Bernoulli equation by accounting for the complete velocity profile, instead of a single peak velocity value, at a given aortic cross section. It thus neglects the contribution of the proximal velocity at the inlet, and it conceptually measures the pressure required to accelerate the blood observed at the given cross section from an idealised static status.

The aortic reservoir function is associated with the change in volume due to the cyclic distension and recoil of the vessel and can be estimated by its elastic module *E*. Pulse wave velocity (*PWV*) is a recognised predictor of *E*, based on the concept that *E* has a direct effect on the speed at which a pressure waveform travels along the vessel. *PWV* was obtained by dividing the distance travelled by a flow waveform by the moving average of the foot-to-foot time between two locations along the aorta and then used to derive *E* using the Moens-Korteweg equation (SM2, Supplementary Material) [18, 19].

### Statistical Analysis

Continuous variables are presented as mean ± standard deviation (SD). The Shapiro-Wilks test was performed on all metrics to test for normal distribution. Comparisons of mean values were performed using Student’s *t*-test and the Mann–Whitney *U*-test as appropriate for the normality of the distribution for each variable. All analyses were undertaken in MATLAB (MathWorks Inc., Natick, MA, USA).

## Results

The conduit and reservoir function of the two cohorts are reported, without any access to ground truth values. Evaluation of proposed method is thus based on a construct validity exercise, where an impaired reservoir and normalised conduit functions are expected in the AA and TA of the HLHS group compared to the controls.

### Largest Anatomical Differences in AA and TA

The main anatomical differences between the two groups were observed in the reconstructed AA and TA, where the average diameter and wall thickness were 37% and 27% larger in HLHS than in AM, respectively (Table 2). This difference in size decreased in the descending segments with a mean diameter of DA1 and DA2 being 29% and 20% larger in the HLHS compared to the AM group, respectively (*p* < 0.01). The mean curvature in the neo-aortas was 36% lower than that in the non-reconstructed ones (*p* < 0.01).

**Table 2:** MRI-derived anatomical characteristics

	HLHS (*n* = 10)	AM (*n* = 6)	*p* value
Diameter (mm/m^2^)			
AA	28.0 ± 2.5	20.6 ± 4.7	< 0.01
TA	27.7 ± 2.7	20.1 ± 2.5	< 0.01
DA1	18.7 ± 1.9	14.5 ± 1.3	< 0.01
DA2	15.6 ± 1.6	13.0 ± 0.8	< 0.01
Wall thickness (mm/m^2^)			
AA-TA	3.7 ± 0.9	2.9 ± 0.2	< 0.01
DA1-DA2	2.9 ± 0.6	2.4 ± 0.1	0.08
Curvature (m^−1^/m^2^)			
AA	13.7 ± 5.3	21.5 ± 3.8	0.01
TA	45.0 ± 13.3	40.3 ± 10.4	0.47
DA1	31.3 ± 11.2	22.7 ± 4.2	0.04
DA2	4.6 ± 1.9	4.4 ± 0.7	0.56

Values are in *mean* ± *SD*. *HLHS*, hypoplastic left heart patients; *AM*, age-matched controls; *AA*, ascending aorta; *TA*, transverse arch; *DA1*, upper thoracic descending aorta; *DA2*, lower thoracic descending aorta

### Conduit Function: Thoracic DA Increases Afterload in HLHS

**Fig. 2:**
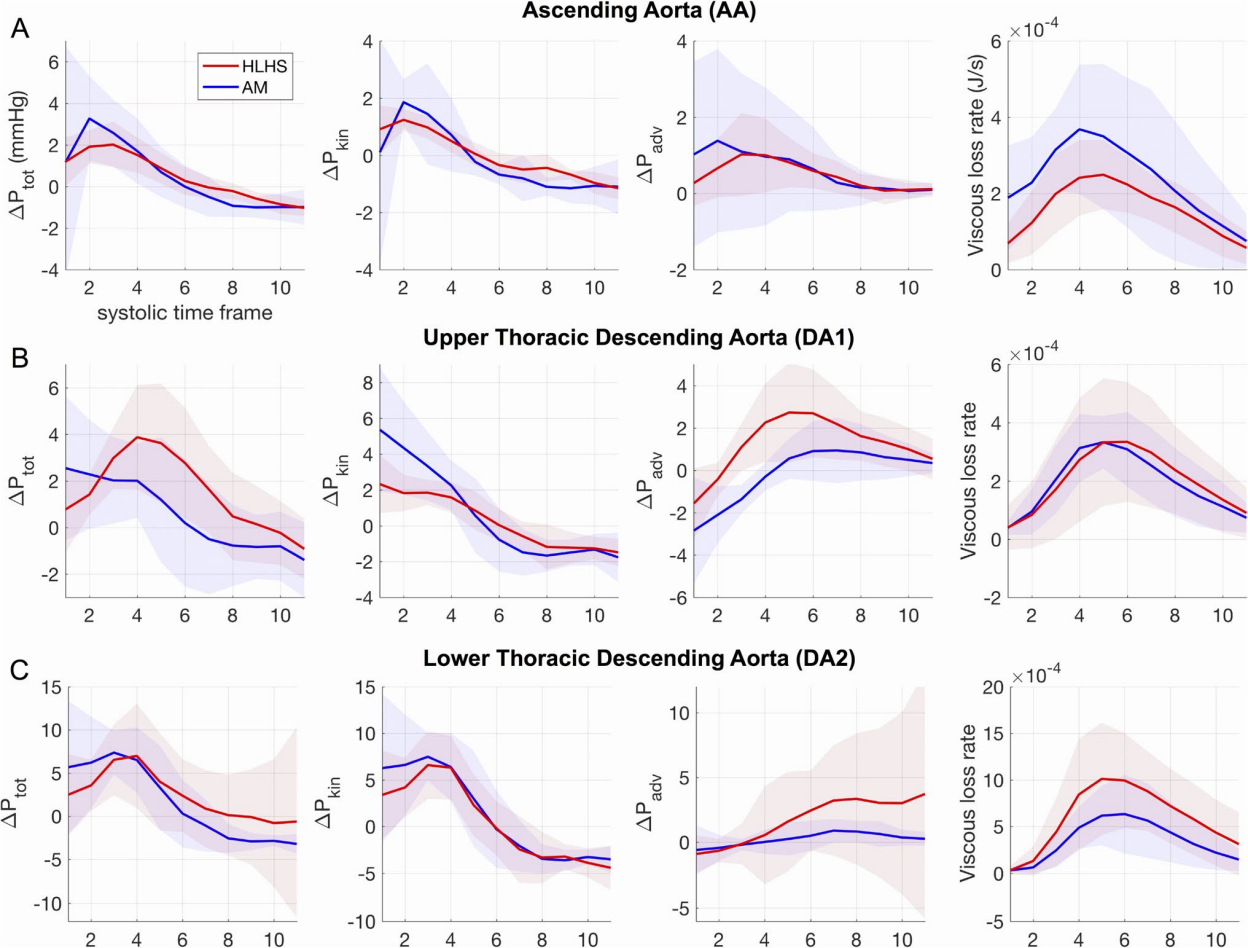
Averaged values by aortic segment for the total (*ΔP*_tot_), kinetic (*ΔP*_kin_) and advective (*ΔP*_adv_) pressure differences and viscous energy loss rate in HLHS and AM groups. A positive pressure difference indicates a larger pressure in the entry than in the exit plane of a segment, corresponding to a temporal flow acceleration (for *ΔP*_kin_) or to a larger momentum in the exit than in the entry plane (for *ΔP*_adv_). The shaded areas indicate 95% confidence intervals

In the AA, the temporal transients of pressure differences showed a qualitatively similar behaviour in both HLHS and AM groups (Fig. 2A). The main divergence was observed in the peak *ΔP*_tot_ and *V*_e_ rate, which in HLHS was significantly lower compared to AM (Table 3).

**Table 3:** MRI-derived pressure differences and timings

	HLHS (*n* = 10)	AM (*n* = 6)	*p* value
Peak *ΔP*_tot_ (mmHg)			
AA	2.34 ± 0.95	4.17 ± 2.95	0.04
DA1	4.64 ± 2.01	3.22 ± 2.62	0.12
DA2	10.00 ± 4.69	10.22 ± 4.31	0.46
Peak *ΔP*_kin_ (mmHg)			
AA	1.50 ± 0.41	2.88 ± 1.24	< 0.01
DA1	2.77 ± 1.25	5.42 ± 3.41	0.02
DA2	8.64 ± 2.89	10.33 ± 4.07	0.17
Peak *ΔP*_adv_ (mmHg)			
AA	1.29 ± 0.98	2.17 ± 2.36	0.15
DA1	3.46 ± 2.08	1.35 ± 1.38	0.02
DA2	4.73 ± 6.72	1.23 ± 0.99	0.11
Viscous energy loss peak (mJ/s)			
AA	0.27 ± 0.09	0.40 ± 0.02	0.03
DA1	0.38 ± 0.21	0.39 ± 0.09	0.56
DA2	1.21 ± 0.50	0.74 ± 0.35	0.03

Values are in *mean* ± *SD*. *ΔP*_tot_, total pressure difference; *ΔP*_kin_, kinetic pressure difference; *ΔP*_adv_, advective pressure difference; *HLHS*, hypoplastic left heart patients; *AM*, age-matched controls; *AA*, ascending aorta; *DA1*, upper thoracic descending aorta; *DA2*, lower thoracic descending aorta

**Fig. 3:**
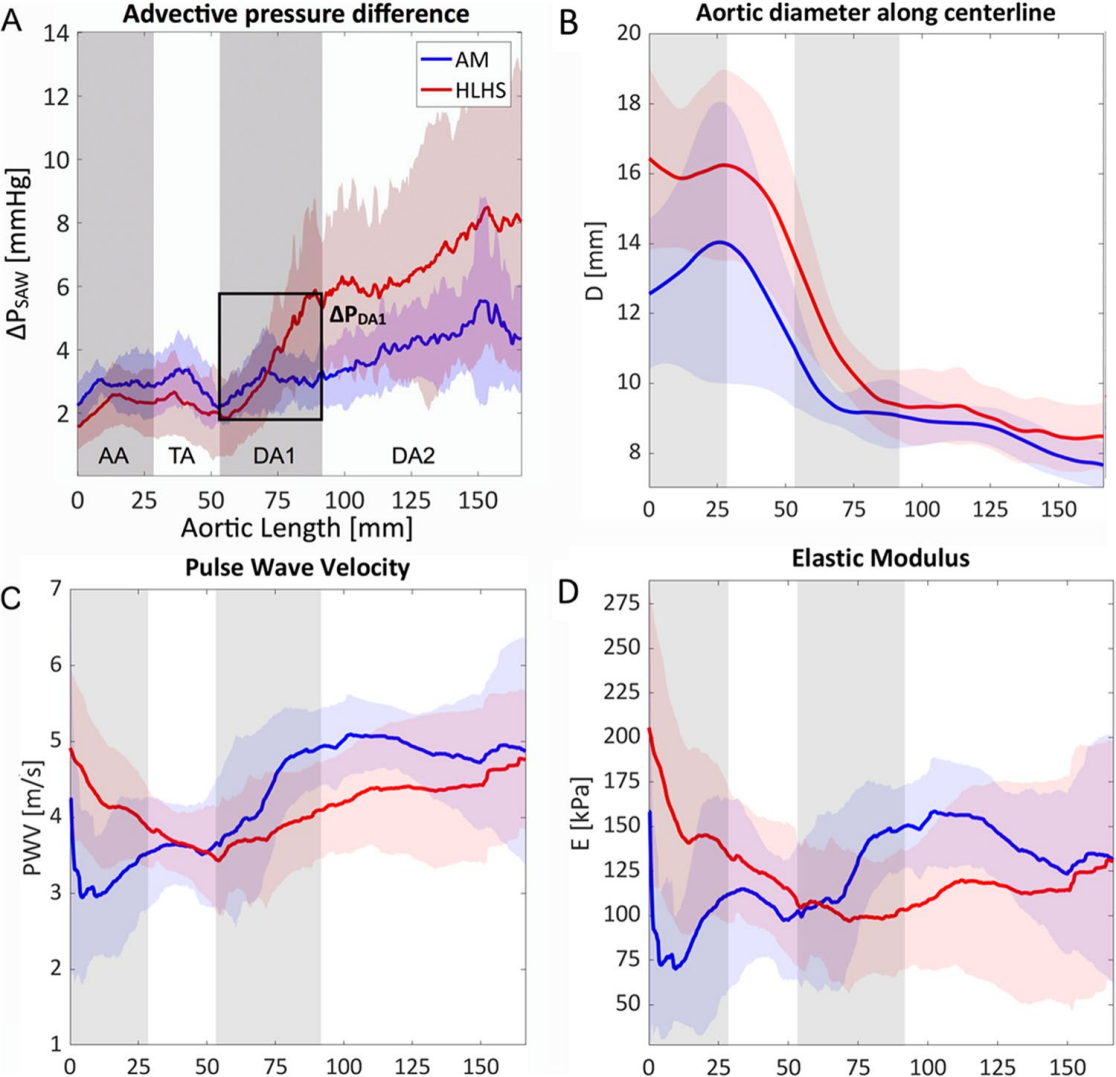
Spatial variations between aortic root (0 mm) and mid-DA2 (~ 160 mm) in peak systolic advective pressure differences (**A**) and diameter (**B**) for conduit function analysis and in pulse wave velocity (**C**) and elastic module (**D**) for reservoir function analysis. The shaded areas indicate 95% confidence intervals

The HLHS DA1, however, showed a significantly larger advective pressure component throughout systole than the corresponding segment of AM (Fig. 2B), with 2.5 times higher peak *ΔP*_adv_ (*p* = 0.02, Table 3). This resulted in a 44% increase in the peak *ΔP*_tot_ of HLHS patients. The spatial analysis provided by *ΔP*_SAW_ at peak systole confirms that the increase in flow momentum mainly originates along the DA1 segment (Fig. 3A): the HLHS group showed a three-fold increase in the advective pressure difference along the DA1 compared to the corresponding mean values in AM (*ΔP*_DA1_ = 3.34 ± 2.11 mmHg vs 1.09 ± 1.05 mmHg, *p* = 0.005).

The momentum of blood created along DA1 was then sustained, and slightly incremented, along the DA2 due to gradual vessel tapering (Fig. 3A, B). This latter segment showed a qualitatively similar behaviour in both groups, but its *ΔP*_adv_ progressively increased in HLHS from mid-systole (Fig. 2C). This segment also exhibited a 63% larger peak of *V*_e_ rate in HLHS despite a small difference in diameter between groups (Table 2).

### Reservoir Function: the HLHS Aorta Is Stiffer in the AA but More Compliant in the DA

In the AA, *PWV* and *E* were higher in HLHS patients than those in in AM controls (Fig. 3C–D). On average, *E* displayed a 69.7% increase in this AA segment. In contrast, the DA1 and DA2 were on average 18.4% more compliant in HLHS (*E* of 111.5 ± 40.8 vs 136.6 ± 29.6 kPa, *p* = 0.07). Despite the TA in HLHS also including homograft tissue, its mean elastic module was similar to AM subjects. The TA, with a lesser extent of homograft tissue, was the segment that displayed a qualitatively similar reservoir function in both groups and acted as transition between AA and DA (Fig. 3D).

The time to peak *ΔP*_tot_ in the AA and DA1 of HLHS subjects was significantly longer than that in AM, as is the duration of the systolic acceleration phase, quantified by the time to peak and to zero-crossing in *ΔP*_kin_ (Table 4). This trend was consistently observed in all aortic segments and indicated a longer early systolic flow acceleration.

**Table 4:** Reservoir function metrics

	HLHS (*n* = 10)	AM (*n* = 6)	*p* value
PWV (m/s)			
AA	4.3 ± 0.8	3.2 ± 0.9	0.04
TA	3.7 ± 0.4	3.6 ± 0.6	1.00
DA1	3.8 ± 0.6	4.3 ± 0.8	0.12
DA2	4.4 ± 0.8	4.9 ± 0.6	0.18
E (kPa)			
AA	154.6 ± 51.5	91.1 ± 44.9	0.04
TA	123.1 ± 31.2	107.4 ± 35.2	0.50
DA1	101.7 ± 28.2	125.0 ± 36.1	0.26
DA2	116.5 ± 53.8	142.5 ± 29.9	0.12
Time to peak *ΔP*_tot_ (ms)			
AA	89.0 ± 31.5	64.3 ± 14.6	0.04
DA1	136.8 ± 43.3	68.5 ± 36.2	< 0.01
DA2	116.6 ± 56.5	86.9 ± 35.6	0.13
Time to peak *ΔP*_kin_ (ms)			
AA	73.9 ± 23.1	60.5 ± 15.9	0.11
DA1	69.3 ± 32.0	62.7 ± 26.8	0.33
DA2	89.0 ± 39.2	73.1 ± 30.0	0.20
Time to zero-crossing *ΔP*_kin_ (ms)			
AA	153.8 ± 39.5	126.9 ± 23.2	0.07
DA1	172.4 ± 41.7	147.1 ± 14.8	0.09
DA2	175.5 ± 42.3	157.4 ± 22.6	0.17

Values are in *mean* ± *SD*. *PWV*, pulse wave velocity; *E*, elastic modulus; *ΔP*_tot_, total pressure difference; *ΔP*_kin_, kinetic pressure difference; *HLHS*, hypoplastic left heart patients; *AM*, age-matched controls; *AA*, ascending aorta; *TA*, transverse arch; *DA1*, upper thoracic descending aorta; *DA2*, lower thoracic descending aorta

## Discussion

A non-invasive assessment of the conduit and reservoir function along the infant aorta is feasible. The neo-aortas in HLHS achieve a normalised conduit function at the cost of reservoir function in the AA segment. Our results identify the DA segments as the weakest link in the HLHS aorta in patients prior to Fontan due to an impaired conduit function.

### Method Validation

Our results indicate that the use of homograft tissue in the AA generates a stiffness increment that reduces the reservoir function, in agreement with the literature [3, 4]. On the other hand, we report a slight improvement in the conduit function of the reconstructed HLHS AA, as expected since this is the primary objective of the reconstructive surgery. Our *PWV* values (average of 4.0 m/s across segments from Table 4) are lower than the ones reported for 6–9-year-old HLHS patients (4.4 m/s [19, 20]), which agrees with the consensus that *PWV* and stiffness increase with age [19, 20]. These positive results constitute the additional construct validity in the assessment of the reservoir and conduit function of an artery, on top of the existing evidence of the ability to estimate PWV [21] and pressure differences [11, 12] from 4D flow MRI.

The change in elasticity caused by the insertion of the homograft patch, potential residual vessel obstructions and abrupt changes in curvature, diameter or stiffness are all contributing factors to haemodynamic inefficiencies and deleterious increases in ventricular afterload [3, 22–25]. Our method can be used to investigate the interplay between vessel anatomy and function, as illustrated by the novel insights gained in current study of the HLHS reconstructed aorta, and without the additional risks of the invasive catheterised recordings.

### Sustained Conduit, but Impaired Reservoir Function in the HLHS AA

Compared to the normal biventricular anatomy, a systemic RV pumped more flow through the neo-aorta at peak systole (about double in our cohort — SM1, Supplementary Material — note that the cardiac output was similar). Despite this difference, the AA conduit function was improved in the HLHS reconstruction with smaller pressure differences *ΔP*_tot_ along the aorta required to drive the flow in systole (Fig. 2A). Thanks to the surgically enhanced diameter and the reduced curvature, this segment showed not normal but even lower energy loss rate and advective pressure difference (surrogate metrics of conduit function) compared to AM subjects and hence accommodated the increased flow demand without extra inefficiencies.

The systemic RV is also a weaker pump than the LV and thus requires additional time to accelerate this larger flow volume, as shown by the delayed zero-crossing and peak in *ΔP*_kin_ indicating a longer and milder acceleration phase in the reconstructed AA compared to the non-reconstructed one. This result is also consistent with findings that the systemic RV in HLHS patients generates flow waveforms with lower energy compared to the single LV [3] and is a sign similar to the lower inotropy reported with the study of aortic flow in dilated cardiomyopathy subjects [26].

The increased stiffness in the AA in HLHS reduces the impedance mismatch between the neo-aorta and the branch vessels to the upper body, which are normally stiffer than the proximal aorta [27]. In normal subjects, this impedance mismatch causes wave reflection at the carotid arteries, preventing part of the energy stored in the aortic waveform from reaching the brain and microvasculature, which undergoes adverse remodelling when exposed to high-pulsatile flow [27, 28]. An increase in AA stiffness would suggest that in HLHS, this protective function is reduced, potentially introducing risks of ischaemia and cognitive impairment at later stages. While the lower-energy waveform from the systemic RV may mitigate this adverse effect, its impact could be significant in the developing vasculature of very young patients and warrants further investigations.

### HLHS DA Displays Better Reservoir Function, but Extra Conduit Inefficiencies

The DA in HLHS is not hypoplastic during foetal development because it is linked to the ductus arteriosus. However, it must still accommodate the increased flow demand without having been surgically enlarged like the AA. Our results show that this causes an increase in flow resistance at the DA1, whose effect is sustained and increased along the DA2 due to vessel tapering (Fig. 3A–B). The resulting afterload increase is thus not due to a localised shape change, but to a lack of vessel calibre along the entire DA with unfeasible surgical resolution (too challenging augmentation of the complete thoracic DA).

Our findings in HLHS patients with median age of 2.7 years suggest that the DA dilation observed in older patients [29, 30] can be a non-pathological adaptation to remove the functional afterload of a small native vessel, facilitated by its lower stiffness at this stage compared to AM controls. However, approximately half of the HLHS patients imaged at age 6 presented a severe stiffening of the DA linked to excessive dilation, which correlated with an increased stiffness in the reconstructed TA [29, 30]. We therefore postulate that 4D flow analysis could contribute to identifying patients that are more at risk of developing a pathological DA stiffening in later stages and thus support inter-stage risk assessment in these fragile and young cohorts.

### Relationship with Wave Intensity Analysis

The adverse effects of changes in vascular impedance were also observed in wave intensity analysis studies on HLHS patients [3, 23], where the peak forward compression wave (a surrogate measure for the maximum rate of pressure rise) was significantly correlated with the size mismatch between the TA and the proximal DA1. Our results also identify the DA1 as the weakest link in the aortic reconstruction and quantified for the first time the increase in pressure difference postulated by wave intensity analysis. The individualised analysis of transients also reveals the existence of abrupt changes of compliance in the TA-DA1 transition in some cases (SM1, Supplementary Material), adding yet another explanation for the generation of wave reflections at this point.

Wave intensity analysis also showed a significantly higher and earlier reflected wave in patients with a repaired coarctation compared to controls, even though no residual obstruction was reported [31]. This reflected wave was associated with higher ventricular afterload, reversal of the stiffness gradient along the TA (with proximal segments stiffer than distal ones) and higher demand for oxygen and cardiac work [32]. As these conditions are also present in HLHS, a high-amplitude reflected wave might occur in these patients too: our finding of a steep afterload increase at the DA1 provides a mechanistic explanation of where this wave may originate.

### Towards Optimisation of Surgical Strategies for Aortic Reconstruction in HLHS

Enabled by the insights generated by proposed methodology, the rationale is that aortic reconstruction should aim to (1) optimise the balance between conduit and reservoir function and to (2) avoid sharp transitions in the spatial longitudinal variation of these two mechanical functions. While these objectives are not easy to achieve in newborns, where tolerances are very small, the assessment of flow efficiency using 4D flow analysis can be instrumental at follow-up in predicting which patients are likely to undergo adverse remodelling.

Our results suggest that the AA reconstruction during stage I could benefit from a smaller extent of augmentation than in the cohort analysed here. The rationale is that reducing the surgical enlargement in the AA may lead to a conduit function still similar to the non-reconstructed aorta (the AA in our HLHS subjects was slightly better than controls, i.e. there is room for tighter reconstructions) while preserving the reservoir function by keeping a larger proportion of native and compliant tissue. This would also limit the stiffness increase in the neo-aorta and thus preserve the protective impedance mismatch at the carotid arteries, potentially reducing cognitive impairment in the long term [5].

### Limitations

Collecting high-resolution 4D flow data in very young children with severe pathologies is challenging. The complex anatomy in the DKS anastomosis, which can include flow from the native aorta, posed challenges in flow acquisition and post-processing, resulting in a high standard deviation in the *PWV* and stiffness results in this segment (note that further challenges could be expected if an end-to-side anastomosis, instead of a side-to-side, is used for the DKS procedure). Wall thickness estimation suffered from large inter-observer variability (SM3, Supplementary Material) but it did not change the qualitative differences between groups (same conclusions from *PWV* and from *E*).

Patients with Norwood operations have varying degrees of patch material to augment the arch, depending on numerous factors including the size of the native aorta and how far the augmentation is taken to the DA. Furthermore, the long-term material properties of commonly used homograft patches exhibit a high variability. Our results were obtained in subjects who received a patch made of pulmonary homograft material and thus might be different in cases where porcine or bovine pericardium is used due to variations in material properties and behaviour in time. These factors, together with the small sample size of the present study, make that our results do not provide conclusive evidence for procedural guidelines but rather only generate new hypotheses that need to be further tested in larger studies. However, it should be noted that a complex congenital condition such as HLHS is characterised by a high inter-individual variability in both anatomy and function and thus requires a personalised assessment of each individual’s pathophysiology and treatment rather than a population-based approach.

Finally, current spatial resolution underestimates viscous effects [33], and all scans were acquired under general anaesthesia. Therefore, the actual demand of blood flow and associated pressure differences to estimate conduit function may be larger than those reported.

## Conclusions

The analysis of 4D flow MRI can characterise the reservoir and conduit function of the aorta in a fully non-invasive and comprehensive way. We demonstrate that such analysis is feasible in a cohort of exceptionally challenging patients and that it can reveal valuable insights into the design of optimal surgical interventions and the mechanisms that cause future vascular remodelling.

## Clinical Relevance

Non-invasive pressure estimation and flow quantification are powerful tools to assess conduit and reservoir function in the HLHS neo-aorta, where invasive measurements are challenging to obtain.

The surgically enhanced HLHS aorta is able to accommodate the increased flow demand in pre-Fontan HLHS efficiently, while the descending aorta, which cannot be augmented during reconstruction, generates a three-fold increase in afterload. Quantifying this effect, alongside the elastic modulus, could help predict the vascular remodelling and stiffening seen at later stages.

## Supplementary Information

Below is the link to the electronic supplementary material.

Supplementary file1 (DOCX 3479 KB)

## Data Availability

The data underlying this article will be available upon publication in a Figshare repository at 10.6084/m9.figshare.16860259.

## Abbreviations

AA: Ascending aorta
*A*_e_: Advective energy
AM: Age-matched (controls)
DA: Descending aorta, whole
DA1: Descending aorta, upper
DA2: Descending aorta, lower
DKS: Damus-Kaye-Stansel
*K*_e_: Kinetic energy
HLHS: Hypoplastic left heart syndrome
LV: Left ventricle
MRI: Magnetic resonance imaging
PC-MRI: Phase-contrast magnetic resonance imaging
PWV: Pulse wave velocity
RV: Right ventricle
SAW: Simplified advective WERP
TA: Transverse arch
*V*_e_: Viscous energy
WERP: Work-energy relative pressure
