# Supplementary material for: Unlocking the Non-invasive Assessment of Conduit and Reservoir Function in the Aorta: The Obstructive Descending Aorta in HLHS
Source: J Cardiovasc Transl Res. 2022 Feb 23;15(5):1075–85. doi: 10.1007/s12265-022-10221-4 (PMC9622527; doi:10.1007/s12265-022-10221-4)
Supplement: Supplementary file 1 — Supplementary file1 (DOCX 3479 KB) [file 12265_2022_10221_MOESM1_ESM.docx]

Supplemental Material - Functionally obstructive descending segment in the infant reconstructed aorta in HLHS

Adelaide de Vecchi, Alessandro Faraci, Joao Filipe Fernandes, David Marlevi, Hannah Bellsham-Revell, Tarique Hussain, Nidhin Laji, Bram Ruijsink, James Wong, Reza Razavi, David Anderson, Caner Salih, Kuberan Pushparajah, David Nordsletten, Pablo Lamata

SM1 - Additional details on the patient population and individualised analysis

All HLHS and controls details are summarised in Table 1. All 10 HLHS patients had undergone arch reconstruction, and no one required an interstage intervention on their arch. No ventricular outflow tract obstruction was reported and trivial atrioventricular valve regurgitation was observed in 4 patients (regurgitant fraction <10%). In all cases, a homograft patch was used to construct an augmented ascending aorta and transverse arch reaching a counter incision on the descending aorta. The median (IQR) number of months between the arch reconstruction and the pre-Fontan scans that were used for this study was 33.8 (29.1, 41.2) months for the non-hybrid cases and 26.1 (19.4 26.6) months for the hybrid cases.

| **Table 1. Patient Data** |
| --- |
| ***HLHS Group (10)***  *Morphology*  Mitral and aortic atresia (3): median ascending aorta (AA) on initial echo 2.5mm  Mitral stenosis and aortic atresia (3): median AA on initial echo 3.2mm  Mitral and aortic stenosis (4): median AA on initial echo 4.2mm  *Surgical Pathway*  Hybrid – Norwood – hemi-Fontan (1, HT001)  Hybrid – Comprehensive stage 2 (2, HT007-8)  Norwood – hemi-Fontan (7, HT002-6, HT009-10)  *Indication* in all: *routine* pre-TCPC scan  ***Control Group (6)***  Normal heart (1, AM001)  - Indication: to review vessels after stroke  Left arch with aberrant right subclavian artery (1, AM002)  - Indication: dysphagia  Right arch with aberrant left subclavian artery (1, AM003)  - Indication: confirm anatomy in asymptomatic patient  Uhl’s anomaly (1, AM004)  - Previous septectomy, oversewing of the tricuspid valve and hemi-Fontan then RV plication  - Indication: routine pre-TCPC scan  No right AV connection, RV hypoplasia, VSD, usually related great vessels (1, AM005)  - Previous shunt and then hemi-Fontan  - Indication: routine pre-TCPC scan  Mitral atresia, pulmonary atresia, aorta from RV (1, AM006)  - Previous septostomy, arterial shunt and septectomy and then hemi-Fontan  - Indication: routine pre-TCPC scan |
| *Aortic atresia (AA), aortic stenosis (AS), atrioventricular (AV), hypoplastic left heart syndrome (HLHS), mitral atresia (MA), mitral stenosis (MS), right ventricle (RV), total cavopulmonary connection (TCPC), ventricular septal defect (VSD)* |

The AM cohort consists of 6 subjects with different indication of scan who all present an unobstructed native aorta where no surgical reconstruction was necessary. Specifically, 3 subjects were imaged to investigate suspected vascular rings (with negative outcome). The remaining three were identified as borderline univentricular.

To highlight the effect of this heterogeneity in the AM cohort, the SAW curves at peak systole are presented for each individual case in Figure 1A. Interestingly a mild increase in the SAW pressure drop along the DA segment is observed in 2 of the 3 AM subjects with univentricular circulation and not in the bi-ventricular AM subjects (Fig. 1A). This additional afterload might be a consequence of the increase in flow volume (Fig 1B). Given the relatively flat and similar SAW transient of the 3 healthy controls (green lines) along the DA, this finding suggests that the restrictive DA might not be an issue that is unique to surgically reconstructive HLHS aortas only.

The individualised analysis reveals an extremely obstructive DA in one HLHS case, highlighted in black in Fig 1 and reporting a pressure drop required to drive the flow exceeding 15mmHg. This outlier (HT007, Fig.2) has the second highest indexed cardiac output in the HLHS cohort (Fig.1B) and the smallest DA cross-sectional area (Fig1.C), causing the significant deterioration of the conduit function observed in this region (Fig1.A). This subject has an elastic modulus along the DA1 that is lower than the healthy controls (Fig.1E), suggesting that there is still room for further dilation at a likely cost of stiffening, although any discussion is very speculative at this stage.

The individualised analysis also reveal the presence of sharp transitions of reservoir function (PWV and elastic modulus, Fig.1D and Fig.1E) at the point of transition between the TA and DA1 in several univentricular cases, in contrast to the relatively homogeneous and gradual increase of stiffness along the aorta at this point in the 3 healthy controls.

| **A** | **B** | **C** |
| --- | --- | --- |
| 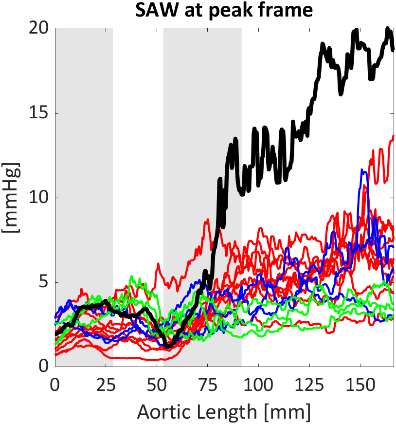 | 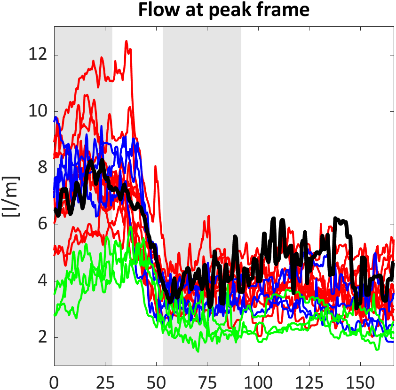 | 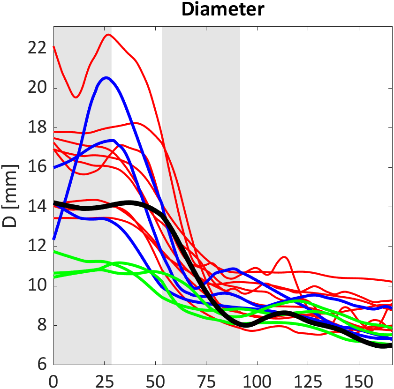 |
| **D** | **E** |  |
| 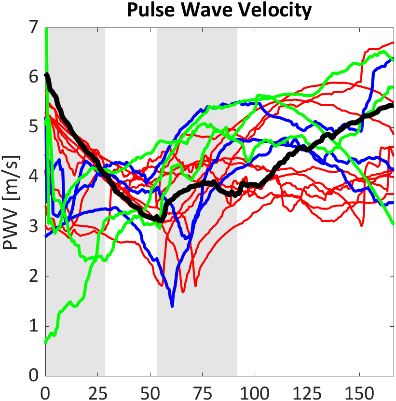 | 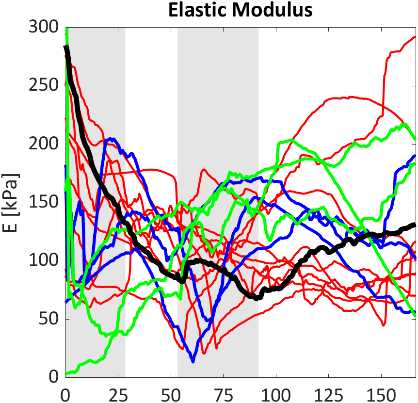 | **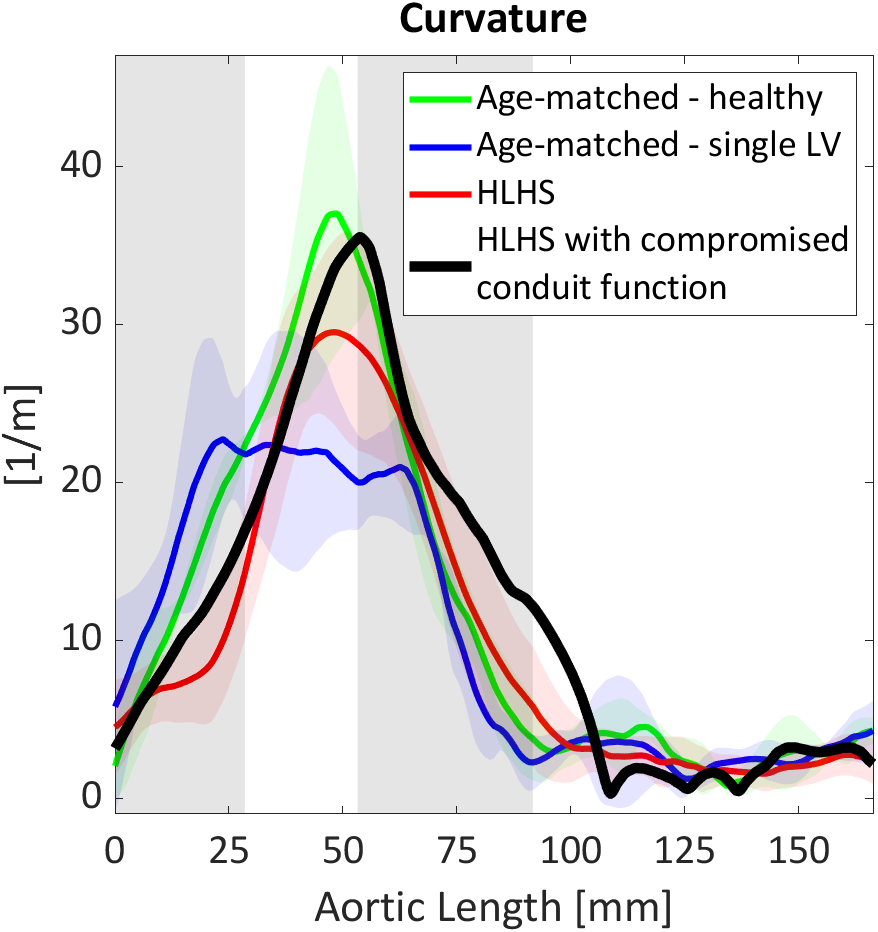** |

**Figure 1.** Individualised analysis across the 16 aortas under study. (A) SAW pressure drop computed at peak systolic frame, (B) flow at peak systolic frame, (C) diameter variations, (D) pulse wave velocity and (E) elastic modulus. The shaded and white areas identify the AA, TA, DA1 and DA2 segments along the aorta. The black line highlights the HLHS patient whose DA conduit function is severely compromised.

| 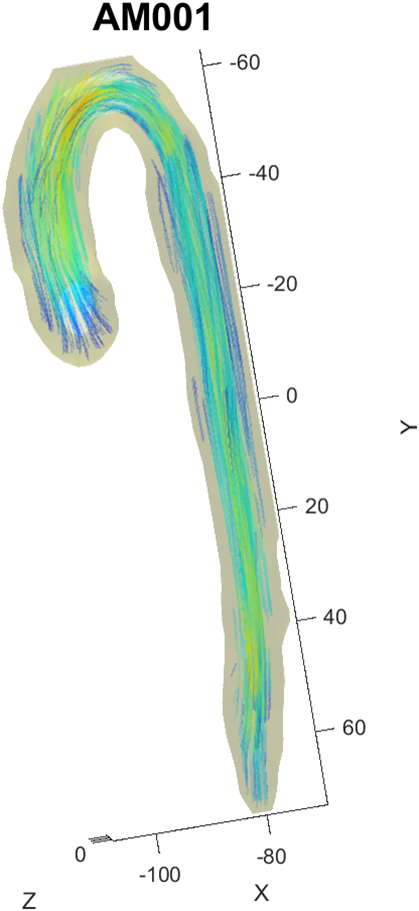 | 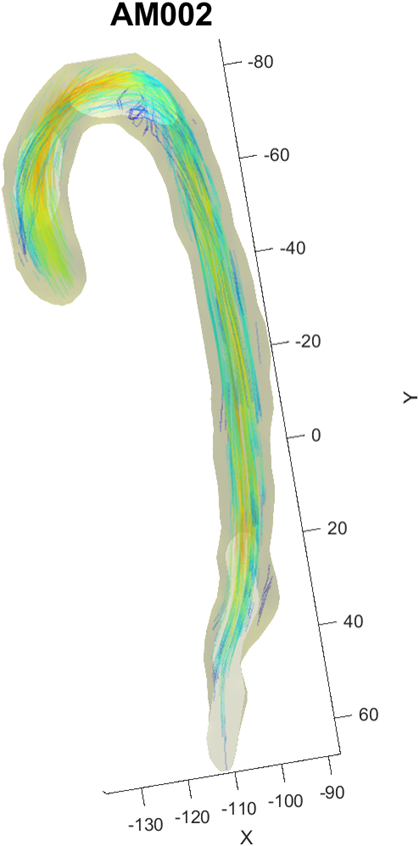 | 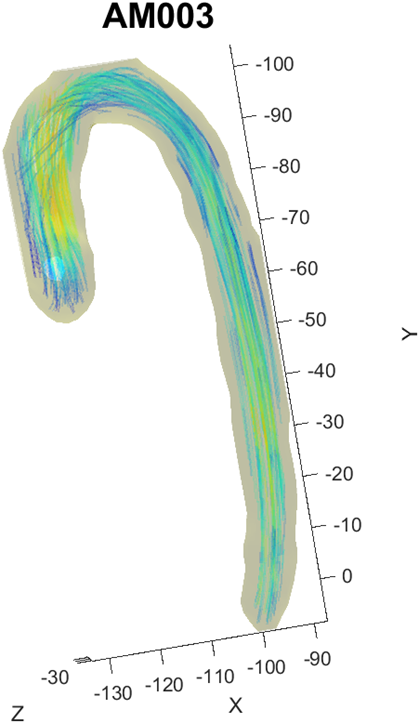 | 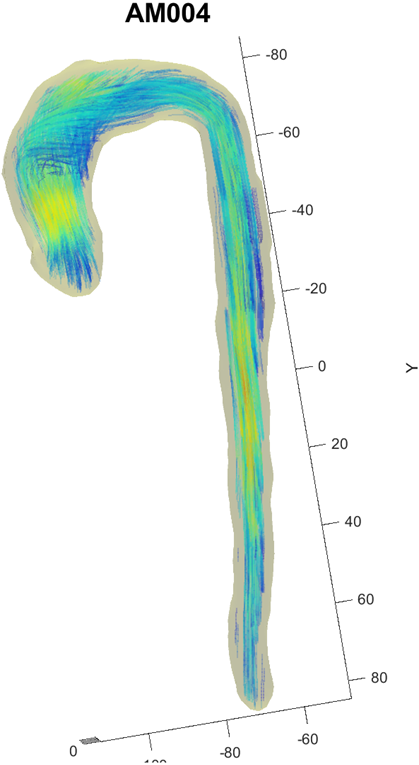 | 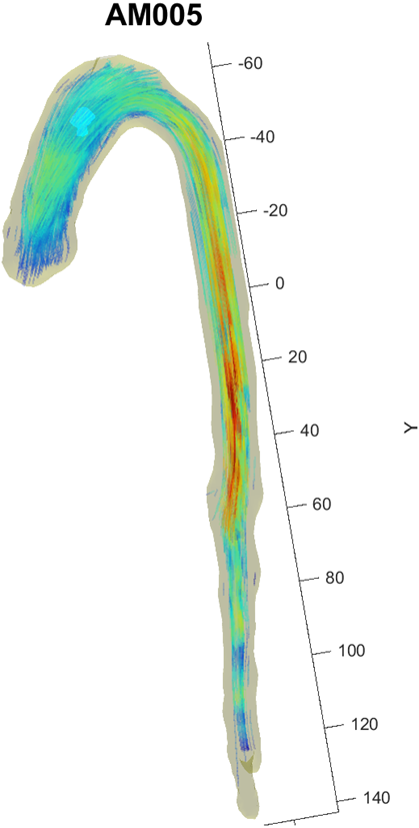 | 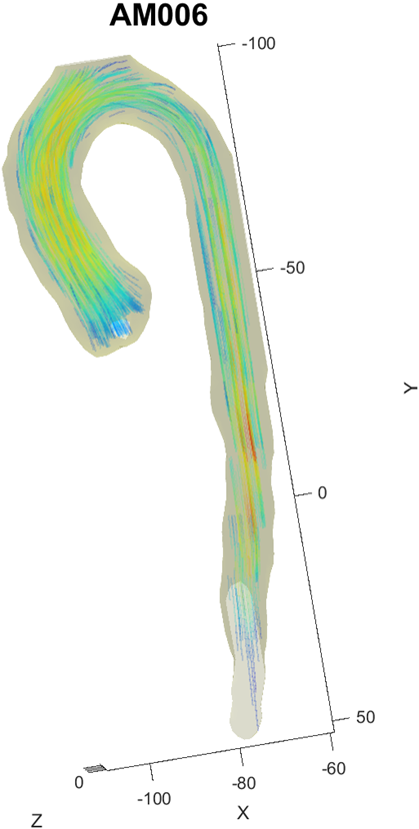 |
| --- | --- | --- | --- | --- | --- |

| 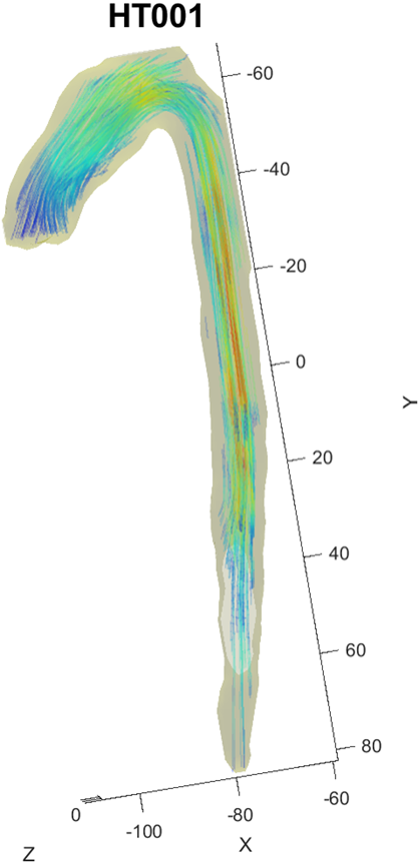 | 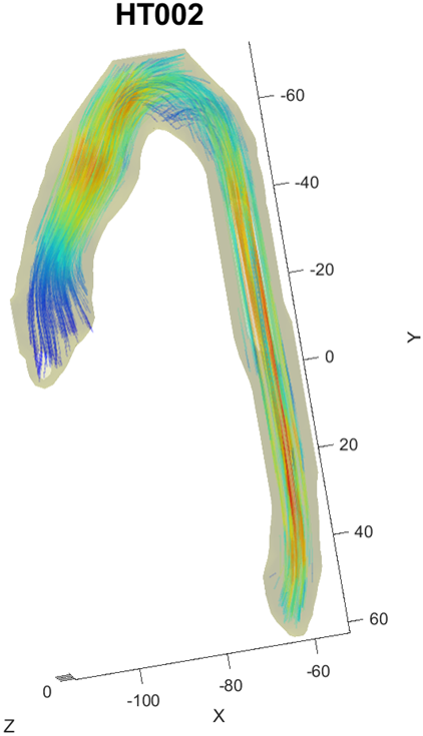 | 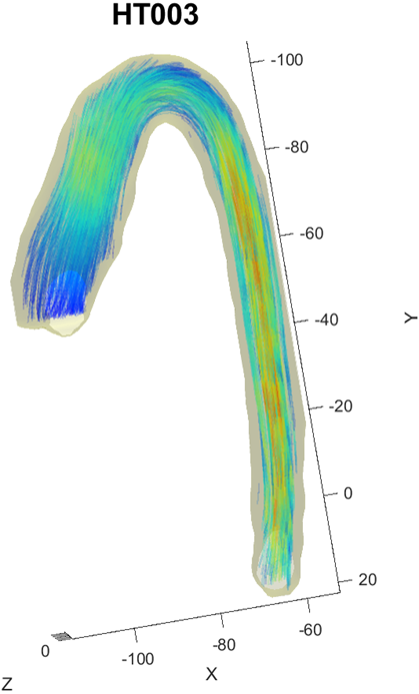 | 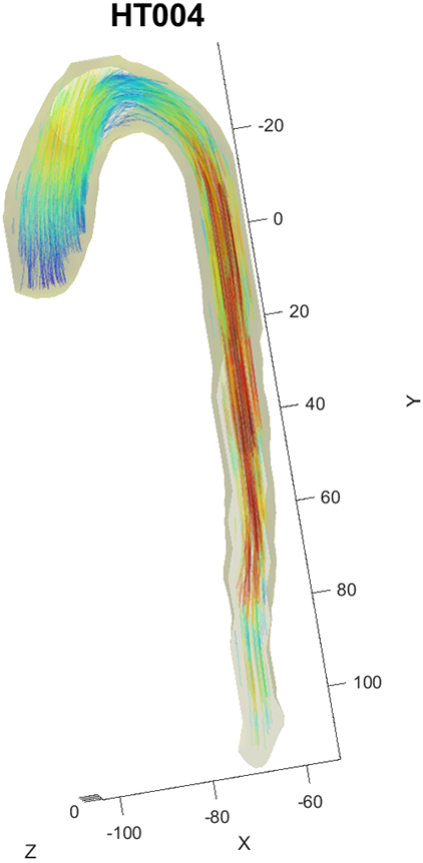 | 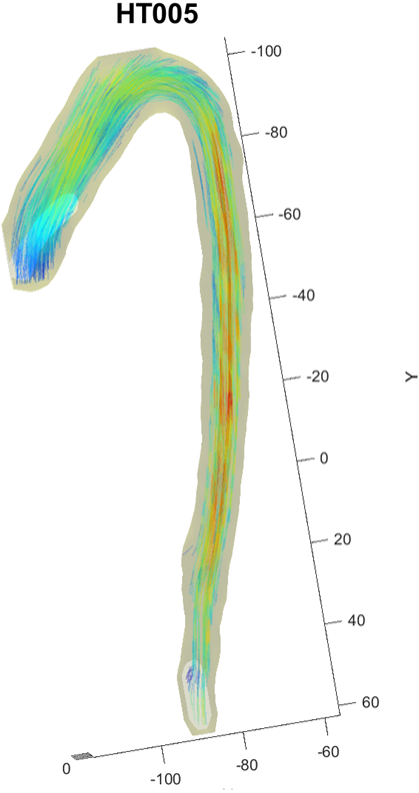 |
| --- | --- | --- | --- | --- |
| 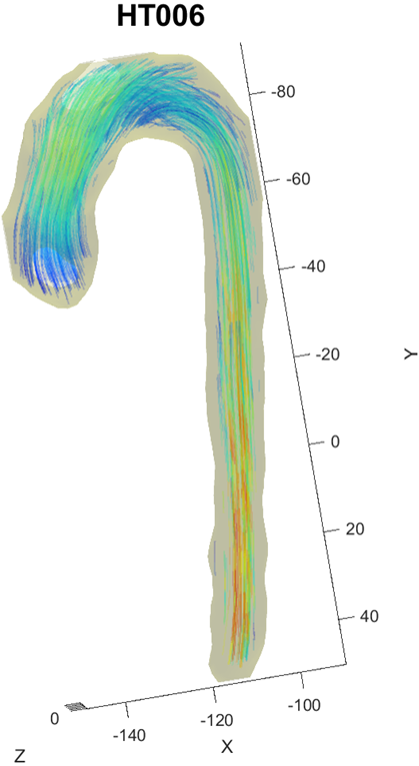 | 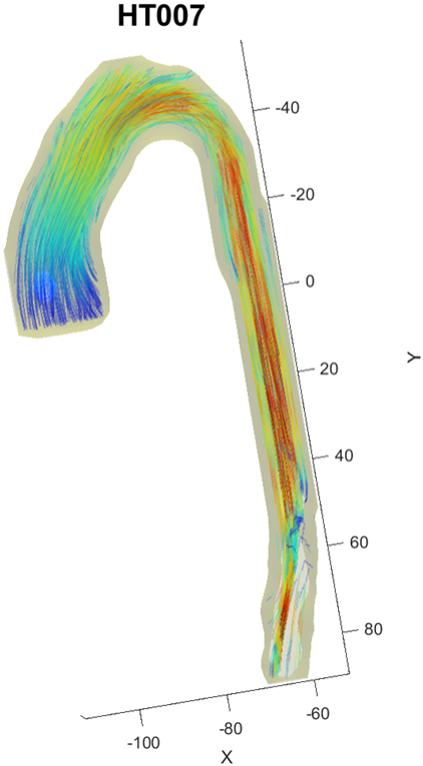 | 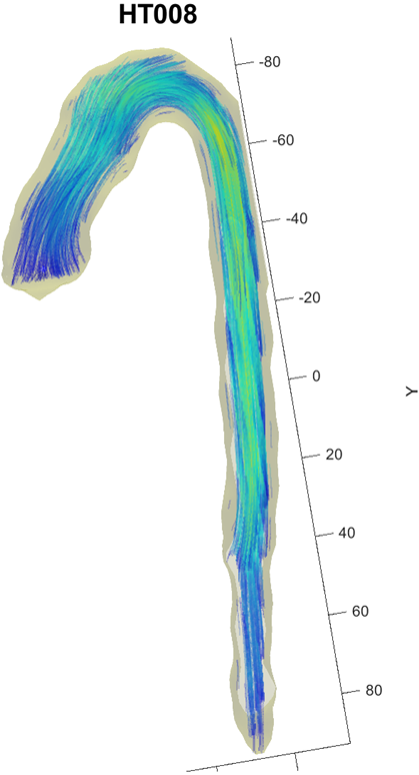 | 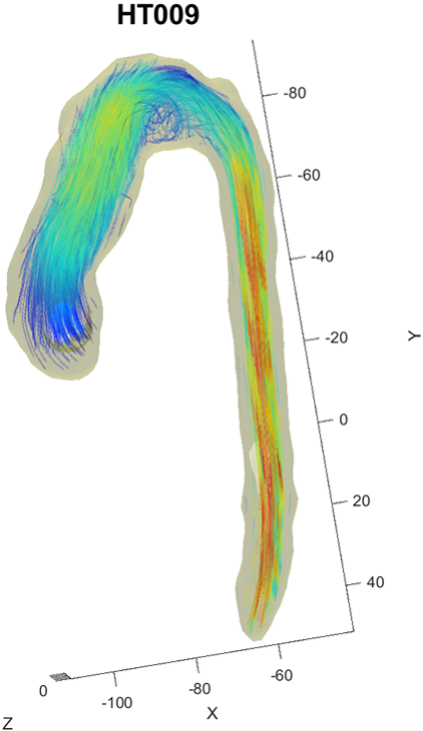 | 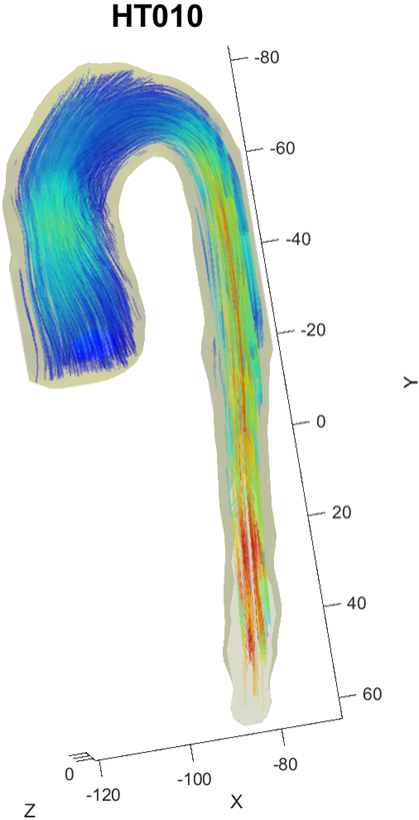 |
| 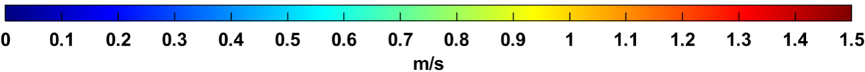 | | | | |

**Figure 2.** Peak flow streamlines across the 16 aortas under study (6 Age Matched, AM001-AM006, and 10 HLHS, HT001 to HT010), colour coded by the velocity magnitude and overlaid on the lumen segmentation obtained from the 4Dflow MRA data.

SM2 - Computation of Pulse Wave Velocity and Elastic Modulus

The pulse wave velocity (PWV) was calculated using the time-to-foot (TTF) methodology with high spatial resolution along the aorta, as enabled by the 4Dflow data.

A collection of aortic perpendicular cross sections, evenly located every 1 mm, is created from the segmentation of the lumen domain. For each cross section of the vessel, a flow temporal waveform is extracted by integrating all the velocity vectors crossing the cross section at each frame, and by fitting a smooth spline curve. In each waveform, the inflection point is computed in the upward part of the curve as the point of maximum gradient during systole, and the foot of the waveform is then obtained as the intersection between the tangent to the inflection point and the horizontal projection of the local curve minimum (Fig. 2A).

After calculating TTF for all the cross sections along the length of the aorta, all time delays from the first point of the centreline are computed (Fig. 2B). From this data, the PWV can then be calculated at each location along the centreline by comparing the distance travelled at a fixed temporal duration $\delta t$. The wider the $\delta t$, the larger the spatial averaging and robustness of the computation. More specifically, the steps are:

- Given the Point $A$ on the centreline and its corresponding TTF_A_ value are considered.
- Find Point $B$ on the centreline as the first one that as TTF_B_ = TTF_A_ + $\delta t$.
- The PWV is calculated as the ratio between the length travelled during $\delta t$ and $\delta t$, i.e.

$${PWV=\overline{AB}}/{\delta t}$$

- The PWV estimate is assigned to all points within the segment AB.
- The same process is repeated for all the points on the centreline until the last one for which another point on the centreline after one $\delta t$ can be found. The process is then repeated backwards starting from the last centreline point.
- As a result, each point of the centreline has several noisy estimates of PWV: the final PWV at each spatial location is then computed as a robust mean of these estimations (i.e. removing the positive skewed velocities greater than a V_max_).

In this solution there are two parameters, $\delta t$ and V_max_, that need to be defined. The criteria chosen for fixing them is that the expected distribution of PWV is not skewed, finding a $\delta t$ of a 55% of the time step of the MRI acquisition and V_max_ = 8.5m/s.

Finally, the incremental elastic module E is then derived from the PWV values using the Moens-Korteweg equation:

$$PWV= \sqrt{\frac{E\cdot h}{2\rho{\cdot r}_{i}}}$$

where h is the aortic wall thickness, ri the internal cross-sectional radius (both measured from MRI data), and ρ the mass density of blood, which is assumed constant at 1060 kg/m3.


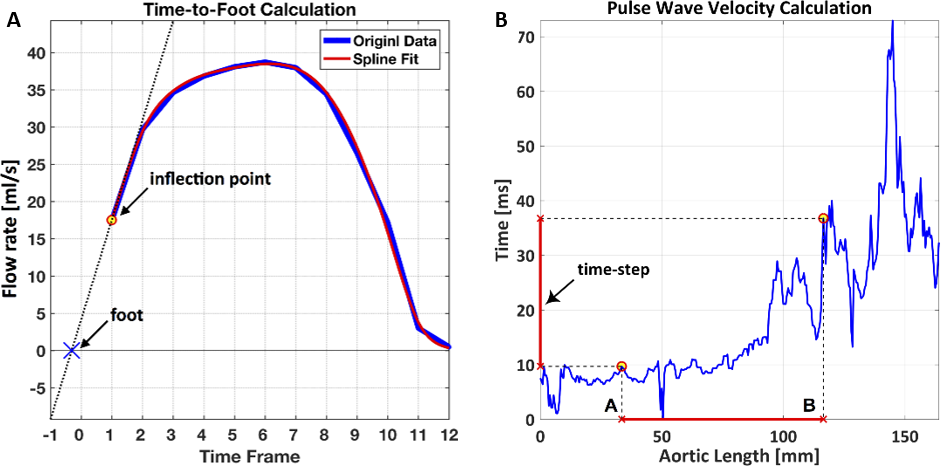


**Figure 2.** (A) Method to calculate time-to-foot (TTF) for each flow wave along the aortic centreline; (B) Plot of TTF at each centreline point along the aorta, with a wide time-step .

SM3 - Study of inter-observer variability in the assessment of wall thickness

The estimation of the thickness of the aortic wall from the 3D SSFP anatomical image is a challenging task. It has been previously used by previous researchers to characterise the infant aorta in HLHS (Voges et al. J Am Heart Assoc. 2015;4:e002107). In this section we report the large inter-observer variability we experienced in its assessment.

Three different observers with experience on cardiovascular MRI analysis were instructed to identify the inner and outer layers of the aortic wall. They were then blindly presented with 32 pre-selected slices so that they were all consistent across all observers, and they were asked to contour the inner and outer layers of the aortic wall. The 32 slices corresponded to the mid ascending and mid descending segments, taken at the level of the pulmonary artery. The interobserver difference was 0.14mm (0.30/0.83; ICC=0.60) in the AA and 0.34mm (0.06/0.69; ICC=0.38) in the DA1.

SM4 - Automatic estimation of curvature

The estimation of aortic curvature was done in a fully automatic fashion from the segmentation mask obtained from the virtual angiography at peak systole (panel B in Figure 1 of main manuscript). The procedure consisted of two steps: the extraction of the centreline of the vessel mask and the estimation of curvature at each point of the centreline.

The centreline was generated by the skeletonization of the vessel mask and the fitting of a smooth cubic function that included all skeleton points. As a result, we have a parametric curve that can be sampled at any desired resolution.

We then computed curvature as the inverse of the radius of the circumference defined by three consecutive points along the centreline, when points were sampled at 1 mm of distance between them.
